# Analysis of overall survival in a large multiethnic cohort reveals absolute neutrophil count of 1,100 as a novel prognostic cutoff in African Americans

**DOI:** 10.18632/oncotarget.8996

**Published:** 2016-04-26

**Authors:** Ioannis Mantzaris, Yiting Yu, Pavlos Msaouel, Anthony P. Lam, Murali Janakiram, Ellen W. Friedman, Ulrich Steidl, Amit K. Verma

**Affiliations:** ^1^ Department of Oncology, Albert Einstein College of Medicine, Montefiore Medical Center, Bronx, New York, USA; ^2^ Department of Medicine, Albert Einstein College of Medicine, Jacobi Medical Center, Bronx, New York, USA; ^3^ Department of Oncology, Stanford University, Stanford, CA, USA

**Keywords:** neutropenia, survival, African American, ethnicity, race

## Abstract

Although absolute neutrophil counts (ANC) below 1.5x10^3^/uL are used to define neutropenia as a marker of increased susceptibility to infections, their relationship with survival has not been examined. Since low counts trigger extensive investigations, determining prognostic cutoffs especially for different ethnicities and races is critical.

A multiethnic cohort of 27,760 subjects, 65 years old and above, was utilized to evaluate the association of neutropenia with overall survival in different ethnicities and races.

The mean ANC was 4.6±1.51x10^3^/uL in non-Hispanic whites, 3.6±1.57x10^3^/uL in non-Hispanic blacks and 4.3±1.54x10^3^/uL in Hispanics (p<0.001). An ANC below 1.5x10^3^/uL was associated with significantly shorter overall survival among whites (HR 1.74; 95% CI 1.18 - 2.58; p<0.001), but not in blacks (HR 0.89; 95% CI 0.86 - 1.17; p=0.40) or Hispanics (HR 1.04; 95% CI 0.76 - 1.46; p=0.82), after adjustment for age, sex, comorbidities, anemia and thrombocytopenia. Using Cox regression multivariable models, an ANC below 1.1x10^3^/uL in blacks was found to be associated with increased mortality (HR 1.86; 95%CI 1.21 - 2.87; p<0.01). We found no association between neutropenia and mortality at any ANC cutoff in elderly Hispanics. In conclusion, neutropenia was found to be an independent prognostic variable in the elderly, when determined in race-specific manner. Most importantly, a cutoff of 1.1x10^3^ neutrophils/uL may be a more prognostically relevant marker in elderly blacks and could serve as a novel threshold for further evaluation and intervention in this population.

## INTRODUCTION

Neutrophils constitute the majority of circulating white blood cells and are crucial components of the innate immunity against bacteria and fungi. An absolute neutrophil count (ANC) of less than 1.5 x 10^3^ cells per microliter (uL) has been adopted by the National Cancer Institute and has been, subsequently, established in clinical practice as the defining cutoff for neutropenia [1]. However, this definition of neutropenia, as a risk factor for infection, originating from leukemia studies in mid-1960s [2], has not been sufficiently studied in the different ethnicities.

Racial and ethnic differences in the distribution of circulating neutrophils were first observed as early as 1941 by Forbes [3]. This observation was further confirmed over the following years [4–6]. Terms, such as “benign ethnic neutropenia”, “benign familial neutropenia” or “pseudo-neutropenia”, were introduced to describe the presence of mild to moderate neutropenia in individuals of African descent, as well as of certain Middle Eastern ethnic background, in the absence of an identifiable cause and a history of recurrent or severe infections [5, 7]. Individuals with the so-called ethnic neutropenia are shown to have a morphologically and functionally normal bone marrow and are not considered being at higher risk for infections or poor infection outcomes [8–11]. Neutropenia in these groups seems to be the result of fewer mature neutrophils exiting, at steady state, the bone marrow's marginal pool [8, 9]. A polymorphism in the Duffy Antigen Receptor for Chemokines (DARC) gene was recently linked to low neutrophil counts in individuals of African ancestry. The mechanism is not fully elucidated, but appears to be associated with a regulatory control over the level of circulating chemokines that, in turn, regulate neutrophil release from the bone marrow [12–14]. Consequently, although a neutrophil count of 1.5 x 10^3^/uL has been conventionally considered the threshold below which increased susceptibility to infections is incurred, this may not be accurate across the different ethnic and racial groups. Moreover, the association between neutrophil count and morbidity or mortality risk may not be mediated by infection alone [15–18].

There are no large studies that have examined the relationship of low neutrophil counts with overall survival. It was, therefore, the aim of this study to 1) look at the association of racial/ethnic variations in neutrophil counts with mortality risk and 2) determine a race/ethnicity-specific lower limit for circulating neutrophils.

With a primary hypothesis that the association of neutropenia, as currently defined, with overall survival differs between Caucasians and individuals of African ancestry, we wanted to further explore for a new race-specific neutrophil cutoff that would better identify the clinically important cases of neutropenia. We focused on subjects above the age of 65 years, an age population frequently seen with cytopenias, and at the same time, particularly vulnerable to their negative effects [19, 20].

## RESULTS

### Absolute neutrophil counts below 1.5 x 10^3^ neutrophils/uL are associated with adverse survival in the non-Hispanic white cohort

**Figure 1:**
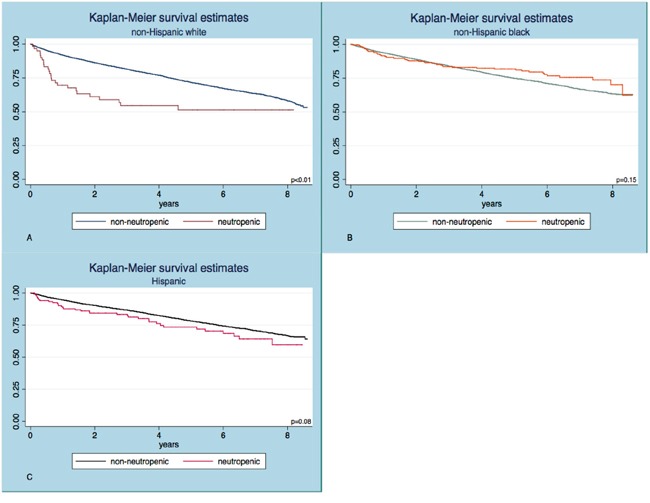
Kaplan Meier survival curves in subjects with and without neutropenia The two curves separate significantly in the non-Hispanic white **A.** but not in the non-Hispanic black **B.** or Hispanic **C**. cohorts. The log-rank test was used to test statistical significance.

The baseline characteristics of elderly individuals with and without neutropenia within each ethnic group are summarized in Table 1. The mean absolute neutrophil count (ANC) was 4.6 ± 1.51 x 10^3^/uL in non-Hispanic whites, 3.6 ± 1.57 x 10^3^/uL in non-Hispanic blacks and 4.3 ± 1.54 x 10^3^/uL in Hispanics (p<0.001). Neutropenia was prevalent in 3.0% of blacks, 0.7% of whites and 1.4% of Hispanics. Notable proportion of blacks (134/276) and Hispanics (72/136) with ANC<1.5 x 10^3^/uL had isolated neutropenia, which was less frequent in whites (16/61). The unadjusted survival experience of neutropenic and non-neutropenic subjects in the different ethnic subgroups is shown in Figure 1; a significantly shorter overall survival (OS) in neutropenics was shown only for whites (p<0.01). The median observation time was 3.7 years in whites (IQR 1.7 – 6.2), 3.2 years in blacks (IQR 1.6 – 5.8) and 3.3 years in Hispanics (IQR 1.6 – 6.0).

**Table 1:** Baseline characteristics of neutropenic and non-neutropenic subjects in each of the three categories of combined ethnicity and race*

	non-Hispanic white (n= 8,649)		*p-value*	non-Hispanic black (n= 9,267)		*p-value*	Hispanic (n= 9,844)		*p-value*
	Neutropenia (n)								
	Yes (61)	No (8,588)		Yes (276)	No (8,991)		Yes (136)	No (9,708)	
	Median (IQR)								
Age, yrs	73 (66-80)	75 (69-80)	*0.18*	70 (66-76)	71 (67-77)	*<0.01*	70 (66-77)	71 (67-76)	*0.51*
CCI	4 (2-8)	4 (2-7)	*0.61*	4 (2-7)	5 (2-8)	*0.02*	3 (1-6)	4 (1-7)	*0.27*
Sex, Male (%)	55.7	46.1	0.13	36.6	33.2	*0.24*	40.4	35.7	*0.25*
Anemia (%)	62.3	27.9	*<0.001*	39.9	38.5	0.64	33.8	24.9	0.02
Thrombocytopenia (%)	52.5	8.8	*<0.001*	23.2	6.4	<0.001	31.6	6.5	<0.001

* CCI: Charlson Comorbidity Index, IQR: Interquartile Range

In multivariable analysis, neutropenia continued to be a significant predictor of higher mortality risk for whites (HR 1.74; 95% CI 1.18 - 2.58; p<0.001), independent of age, sex, comorbidities, anemia and thrombocytopenia. On the contrary, it was associated with a lower, albeit statistically non-significant risk in blacks (HR 0.89; 95% CI 0.86 - 1.17; p=0.40) and was not significantly associated with decreased overall survival in Hispanics (HR 1.04; 95% CI 0.76 - 1.46; p=0.82), adjusted for the same covariates. In all three groups, age, male sex, Charlson Comorbidity Index (CCI), anemia and thrombocytopenia were independent predictors of mortality (Table 2).

**Table 2:** Multivariable Cox PH Models on the association of neutropenia with mortality risk in separate ethnic groups

	non-Hispanic white (n=8,649)	non-Hispanic black (n=9,267)	Hispanic (n=9,844)
	HR (95% CI; p-value)*		
Neutropenia**	1.74 (1.18 - 2.58; <0.01)	0.89 (0.86 - 1.17; 0.40)	1.04 (0.76 - 1.46; 0.82)
Age	1.06 (1.06 - 1.07; <0.01)	1.05 (1.05 - 1.06; <0.01)	1.06 (1.05 - 1.06; <0.01)
Sex**	1.30 (1.20 - 1.41; <0.01)	1.36 (1.24 - 1.49; <0.01)	1.40 (1.28 - 1.54;<0.01)
CCI	1.09 (1.08 - 1.10; <0.01)	1.08 (1.07 - 1.09; <0.01)	1.10 (1.09 - 1.11; <0.01)
Anemia**	1.74 (1.60 - 1.90; <0.01)	1.79 (1.63 - 1.96; <0.01)	2.07 (1.88 - 2.28; <0.01)
Thrombocytopenia**	1.35 (1.20 - 1.53; <0.01)	1.42 (1.23 - 1.64 <0.01)	1.84 (1.60 - 2.11; <0.01)

* HR: Hazard Ratio, CI: Confidence Interval

** the reference category is female sex and absence of neutropenia, anemia or thrombocytopenia

### Lower absolute neutrophil count cutoffs are prognostic in blacks

**Figure 2:**
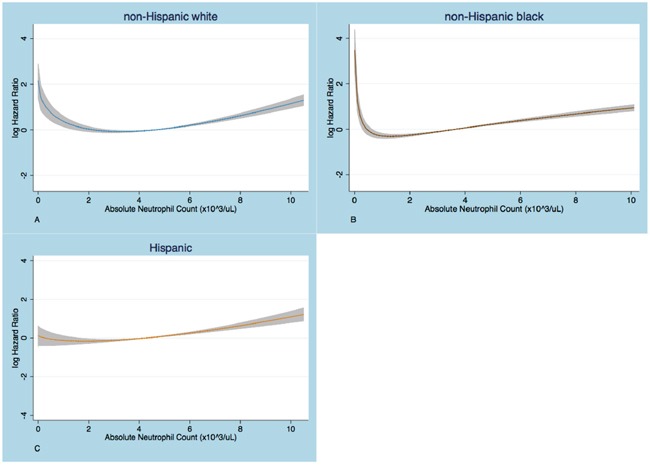
Partial residual plots that show the relationship of log HR with absolute neutrophil count (ANC) The logHR increases below an ANC cut-point that is higher in whites **A.** than in blacks **B**. No substantial increase in logHR at low ANC is seen in Hispanics **C**. The graphs are based on Cox PH regression models on fractional polynomials in ANC, adjusted for age, sex, Charlson comorbidity index, anemia and thrombocytopenia.

Next, we wanted to explore whether neutropenia defined by a different neutrophil count cutoff in non-Hispanic blacks and Hispanics could be associated with shorter survival, similarly to what is shown in whites using the conventional definition. The relationship between mortality risk and ANC was graphically assessed by partial residual plots (Figure 2). In non-Hispanic whites and blacks a U-shaped curve was seen, yet, the ANC cut-point below which the risk of death increased was different and lower for the black compared to the white subcohort. This U-shaped relationship was attenuated in Hispanics.

To determine a prognostic ANC cutoff for African Americans and Hispanics, we used multivariable Cox Proportional Hazard models adjusting for age, sex, anemia, thrombocytopenia and other comorbidities. We observed that neutrophil counts lower than 1.1 x 10^3^ cells/uL were associated with a significantly greater risk of death (HR 1.86; 95%CI 1.21 - 2.87; p<0.01), compared to those with ANC at or above that threshold in the African American cohort. Using this cut-point, black patients with neutropenia were more likely to be anemic and thrombocytopenic too compared to those without neutropenia, similar to what was seen in white subjects with neutrophils below 1.5 x 10^3^/uL (Table 3). We found no neutrophil cut-off in Hispanics at which neutropenia was significantly associated with increased mortality risk.

**Table 3:** Prevalence of cytopenias in neutropenic and non-neutropenic whites and blacks, using conventional and race-specific neutropenia definitions

	non-Hispanic white (n= 8,649)			non-Hispanic black (n= 9,267)					
	ANC <1.5 (61)	ANC ≥1.5 (8,588)	*p-value*	ANC <1.5* (276)	ANC ≥1.5* (8,991)	p-value*	ANC <1.1 (61)	ANC ≥1.1 (9,206)	*p-value*
Anemia (%)	62.3	27.9	*<0.001*	39.9*	38.5*	0.64*	50.8	38.4	*0.048*
Thrombocytopenia (%)	52.5	8.8	*<0.001*	23.2*	6.4*	<0.001*	36.1	6.7	*<0.001*
Anemia & thrombocytopenia, %	41.0	3.5	*<0.001*	11.6	3.1	*<0.001*	24.6	3.2	*<0.001*

* from Table 1

## DISCUSSION

Race is one of the most common contributing factors to the variation of neutrophil counts in adults and has formed the basis of this study [26]. A 4.5% prevalence of neutrophil counts below 1.5 x 10^3^/uL is to date the most representative estimate for African Americans [27]. The recent discovery that lower neutrophil counts (ANC) observed among individuals of African descent are associated with the Duffy Antigen Receptor for Chemokines (DARC) “null” genotype has shed light to the likely racial origin of these differences [12, 13, 28].

Although a benign physiology and the absence of susceptibility to infections, has been well documented in the literature [8-10, 13, 14], the reference range for neutrophils used in clinical practice is the same across races and ethnicities. As such, individuals primarily of African ancestry are diagnosed with neutropenia that often represents the so-called benign ethnic neutropenia, yet frequently undergo extensive hematologic work-up, especially in the elderly. Moreover, ‘abnormally’ low baseline neutrophil counts can influence treatment intensity in cancer, with frequent interruptions or dose modifications ultimately jeopardizing its effectiveness [29, 30]. Finally, a substantial proportion of patients of certain ethnic and racial background with baseline ANC below 1.5 x 10^3^/uL are excluded from clinical trials, further augmenting the under-representation of ethnic ‘minorities’ in cancer research [11].

It was the goal of this present study to look at the association of racial and ethnic variations of neutropenia with mortality risk and ultimately look for a race-specific lower limit of normal for circulating neutrophils. The use of such a cutoff could more accurately identify those individuals of African decent in whom neutropenia is most likely a pathologic manifestation rather than a variation of normal. We used overall survival as the outcome of interest to broadly examine the relationship of neutropenia with overall wellbeing. We decided to look at this association amongst elderly individuals, an age group particularly vulnerable to cytopenias [19, 31, 32]. This study is the first, to our knowledge, report on the survival implications of neutropenia in the elderly, especially in relation to the different ethnicities and races.

Using a large urban multiethnic, multiracial cohort of elderly subjects, we confirmed that neutropenia, defined as ANC below 1.5 x 10^3^/uL, is an unusual finding in non-Hispanic Caucasian (0.7%), but relatively frequent in non-Hispanic black subjects (3.0%). These prevalence estimates are not far from what is reported in the literature by the largest to date study of neutropenia prevalence in the US (0.79% and 4.5%, respectively).[27] We found that using an ANC cutoff of 1.5 x 10^3^ cells/uL, as the established threshold in clinical and research practice, neutropenia has prognostic value for white, but not for black elderly subjects, and this was independent of other cytopenias, age, sex and comorbidity burden. By using this ANC cutoff, 41% of white elderly neutropenic subjects were noted to be pancytopenic, whereas pancytopenia was noted in only 11.6% of their black counterparts. Almost half of black neutropenic subjects had isolated neutropenia. Interestingly, an ANC below 1.5 x 10^3^/uL fell more than 2 standard deviations (SD) away from the mean neutrophil count in the white subcohort, hence appropriately outside a reference range determined by mean ANC ± 2SD, as the one currently in effect. However, it was only 1.6 SD below the mean ANC in the African American subgroup, as blacks were shown to have, on average, significantly lower neutrophil counts than the whites. This finding supports the notion that, had a specific reference range for neutrophil counts in African Americans, been determined, it would include ANC of 1.5 x 10^3^/uL, as a value within the ‘normal’ range.

We, then and most importantly, showed for the first time that it is those neutropenic black elderly individuals with an ANC below 1.1 x 10^3^ cells/uL that have a survival disadvantage, compared to those with higher counts. In our cohort, the majority of blacks labeled neutropenic were noted to have ANC of 1.1 x 10^3^ cells/uL or above, an observation that is in line with the findings of Hsieh et al.[27]. Finally, the cut-point of 1.1 x 10^3^ neutrophils/uL seems to discriminate better those neutropenic blacks with other concurrent cytopenias from those without and hence is more likely to uncover underlying bone marrow pathology. All together, these findings suggest that the “normal” range for ANC currently in use probably overestimates the prevalence of neutropenia in elderly individuals of African decent and that blacks with neutrophil counts above 1.1 x 10^3^ cells/ul could, in reality, be functionally normal.

In our hispanic subcohort, consisted mainly by Puerto Ricans and Dominicans, the prevalence of neutrophil counts below 1.5 x 10^3^/ul (1.4%) was intermediate between that of non-Hispanic whites and blacks. However, Hispanic neutropenics were not identified as a subgroup at significant risk for shorter survival compared to those with higher counts, yet with a relative hazard in-between of what was shown in whites and African Americans. These findings could be interpreted on the basis of the racial heterogeneity of the hispanic ethnicity. This could also explain the different neutropenia prevalence estimate found in our study and that reported by Hsieh et al. for their hispanic group, consisted exclusively of Mexican-Americans with presumably different racial distribution [27].

We found no neutrophil cut-off in Hispanics at which neutropenia was significantly associated with increased mortality risk. It is possible that death events of undocumented subjects were not captured in this dataset in which date of death was obtained from the Social Security Death Index. Given the demographics of this cohort, this more likely affected the hispanic subgroup. Missing death events could have hindered our ability to more accurately determine the relationship of neutrophil counts with mortality risk in this ethnic group. Finally, since ethnic variations in neutrophil distribution are recently shown to be likely of racial origin [12, 13], future studies to explore the appropriateness of the neutropenia definition in Hispanics should ascertain knowledge on the racial composition of a respective hispanic cohort.

There are several limitations in the present study. Blood counts are known to fluctuate during an acute illness or secondary to medications, yet we believe that the exclusion of subjects with recent hospitalizations and primarily the large sample size should have been adequately protective against such effects. The retrospective design prevented the accurate determination of the etiology of neutropenia. However, we took care in our analysis to control for clinical entities, incorporated in the Charlson comorbidity index, likely to influence neutrophil counts. This study, nevertheless, has certain strengths, as well. An ethnically and racially diverse urban outpatient elderly population provided a very large pool of data that facilitated a stratified and multivariable analysis. Although this cohort represents the population of a single institution, it consists of cases encountered in outpatient settings within the various facilities that comprise the Montefiore Medical system, improving the generalizability of the results.

In conclusion, the present study of a large inner city cohort demonstrated that neutropenia could be an independent predictor of mortality in the elderly. However, its prognostic value is likely to vary among different ethnicities and races according to the definition used. In non-Hispanic blacks, setting the ‘lower limit of normal’ for neutrophils at 1.1 x 10^3^ cells/uL could more accurately identify those cases of neutropenia with an underlying pathology, at risk for adverse outcomes. Consequently, this cutoff could serve as a novel threshold for evaluation and intervention in elderly subjects of this racial population. It should be the subject of future studies to confirm our findings in other multiethnic, multiracial cohorts and ultimately extend our understanding of the prognostic implications of neutropenia by ethnicity and race to other age groups.

## MATERIALS AND METHODS

### Study cohort

The study cohort included all individuals at 65 years of age and above that had visited an ambulatory clinic within the Montefiore Medical system in the Bronx, NY, from January 1^st^ 1997 to May 1^st^ 2008 and had a complete blood count drawn within 3 months from the index clinical encounter. To exclude acutely ill patients, those who had been hospitalized within a month from the date of the blood test were removed from the initial sample. Data on demographic and clinical parameters including blood counts and comorbidities were retrieved.

The cohort was generated with the use of Clinical Looking Glass (CLG®) from a multiethnic, multiracial inner-city population of patients. CLG is a software tool developed at Montefiore Medical Center that utilizes integrated clinical, demographic and administrative information to generate datasets accessible to statistical processing. The study was approved by the Einstein Institutional Review Board.

Demographic data (age, sex, ethnicity and race) are based on information obtained at patients' registration. Ethnicity and race were self-reported. Ethnicity was coded as Not Hispanic and Hispanic, while race was reported according to the Office of Management and Budget (OMB) 1997 classification [21]. The Charlson Comorbidity Index (CCI), a well-validated measure of the morbidity burden on prognosis, was calculated in CLG software.[22] CCI is a weighted score that takes into account both the number and the severity of comorbid conditions, and was developed to evaluate their impact on mortality in longitudinal studies. Increased levels of the comorbidity index are associated with stepwise increases in the cumulative mortality; four risk categories were identified in the index's initial description with scores of “0” “1-2” “3-4” and “≥5” [22]. CCI has been adapted for use with ICD-9-CM codes and has been extensively validated in the outpatient setting [23, 24]. The Social Security Death Index was used by CLG to obtain the date of death and mortality data were matched on the patient's name and date of birth.

### Statistical analysis

The initial sample included 36,174 subjects. 3,852 subjects with leukocytosis, erythrocytosis and thrombocytosis, almost exclusively represented in the non-neutropenic subgroup, were excluded. Leukocytosis, erythrocytosis and thrombocytosis were defined, per established criteria, as WBC>11x10^3^/ul, hemoglobin >16.5 mg/dl in women and >18.5 mg/dl in men, and platelet count > 450x10^3^/ul, respectively. There were limited observations for race other than the white and black among the Not Hispanic ethnic group; as such, along with those of unknown ethnicity and race, 4,017 additional individuals were excluded from the analysis. Because of missing data on race for Hispanics, the remaining subjects were categorized into three groups based on composite information on race and ethnicity, referred hereto as ethnic groups: non-Hispanic whites, non-Hispanic blacks (blacks or African Americans) and Hispanics. Due to lack of follow-up time, 545 observations were ultimately excluded from the survival analyses. The final sample was consisted of 27,760 individuals.

The distribution of absolute neutrophil count (ANC) was examined graphically and the mean difference by ethnic groups was compared with one-way analysis of variance (ANOVA). We proceeded to stratified analyses by the three categories. Stratification was based on the a priori assumption of neutropenia effect modification by race. Descriptive statistics compared age, sex, CCI, hemoglobin and platelet counts between neutropenic and non-neutropenic subjects within each separate stratum. Neutropenia was defined as ANC below 1.5 x 10^3^ cells/uL, based on the neutrophil count seen in the complete blood count at the index date for study inclusion. Continuous variables were compared with the Mann Whitney (U) test, due to violations of the normality assumption. Comparison of proportions was done with the Pearson's *χ^2^* test.

The Kaplan Meier survival function was used to depict the unadjusted survival experience of neutropenic and non-neutropenic subjects within each cohort and differences were tested with the log-rank test. Cox Proportional Hazard (PH) multivariate regression models were subsequently constructed to look at the mortality risk associated with neutropenia, adjusted for age, sex, CCI, hemoglobin and platelet count. Those covariates were selected based on a priori determination of their clinically meaningful association with overall survival. The assumption of linearity for continuous and ordinal variables was evaluated with use of fractional polynomials and assessment of post-estimation partial residual plots, as well as with the dummy variable method. Age and CCI were, thus, treated as continuous variables, while hemoglobin and platelet count variables were dichotomized to denote anemia and thrombocytopenia. Anemia was defined, according to the World Health Organization (WHO) criteria of hemoglobin below 12g/dl and 13g/dl in female and male subjects, respectively, while thrombocytopenia was defined as a platelet count below 150 x 10^3^/ul [25]. The PH assumption was assessed graphically with log-log curves and was statistically tested based on analysis of Schoenfeld residuals.

In order to explore for cohort-specific cutoffs for neutropenia, we initially assessed the relationship of mortality risk with absolute neutrophil count graphically, with partial residual plots, adjusted for age, sex, CCI, anemia and thrombocytopenia, in each ethnic group. Subsequently, Cox PH multivariable models, adjusting for the same covariates, were built using serially lower neutrophil thresholds, by decrements of 100cells/ul, till a statistically significant risk ratio was observed. The aforementioned methods were used to evaluate the linearity and PH assumptions.

All tests were two-sided and an alpha 0.05 level was used to determine statistical significance. Statistical analyses were performed in Stata software, version 12.0.

## References

[1] (2010). Common Terminology Criteria for Adverse Events (CTCAE)_v4.03. National Cancer Institute.

[2] Bodey GP, Buckley M, Sathe YS, Freireich EJ (1966). Quantitative relationships between circulating leukocytes and infection in patients with acute leukemia. Ann Intern Med.

[3] Forbes W, Johnson R, Consolazio F (1941). Leukopenia in Negro workmen. The American Journal of the Medical Sciences.

[4] Davis LR, Barnard HF (1967). Leukopenia in West Indians and Africans. The Lancet.

[5] Shoenfeld Y, Weinberger A, Avishar R, Zamir R, Gazit E, Joshua H, Pinkhas J (1978). Familial leukopenia among Yemenite Jews. Isr J Med Sci.

[6] Shaper Ag Fau - Lewis P, Lewis P (1971). Genetic neutropenia in people of African origin. The Lancet.

[7] Shoenfeld Y, Alkan ML, Asaly A, Carmeli Y, Katz M (1988). Benign familial leukopenia and neutropenia in different ethnic groups. Eur J Haematol.

[8] Mason Ba Fau - Lessin L, Lessin L Fau - Schechter GP, Schechter GP (1979). Marrow granulocyte reserves in black Americans. Hydrocortisone-induced granulocytosis in the “benign” neutropenia of the black. Am J Med.

[9] Shoenfeld Y, Aloni D, Keren G, Shaklai M, Djaldetti M, Pinkhas J (1981). Effect of physical effort on the white blood cells in benign familial leukopenia. Acta Haematol.

[10] Ash RC, Mendelsohn LA, Marshall ME (1986). Hemopoietic marrow function in chronic neutropenia of blacks: cure of aplastic anemia by allogeneic marrow transplantation from a neutropenic sibling donor. Am J Hematol.

[11] Hsieh MM, Tisdale JF, Rodgers GP, Young NS, Trimble EL, Little RF (2010). Neutrophil count in African Americans: lowering the target cutoff to initiate or resume chemotherapy?. J Clin Oncol.

[12] Nalls MA, Wilson JG, Patterson NJ, Tandon A, Zmuda JM, Huntsman S, Garcia M, Hu D, Li R, Beamer BA, Patel KV, Akylbekova EL, Files JC, Hardy CL, Buxbaum SG, Taylor HA (2008). Admixture mapping of white cell count: genetic locus responsible for lower white blood cell count in the Health ABC and Jackson Heart studies. Am J Hum Genet.

[13] Reich D, Nalls MA, Kao WH, Akylbekova EL, Tandon A, Patterson N, Mullikin J, Hsueh WC, Cheng CY, Coresh J, Boerwinkle E, Li M, Waliszewska A, Neubauer J, Li R, Leak TS (2009). Reduced neutrophil count in people of African descent is due to a regulatory variant in the Duffy antigen receptor for chemokines gene. PLoS Genet.

[14] Gibson C, Berliner N (2014). How we evaluate and treat neutropenia in adults. Blood.

[15] Weijenberg MP, Feskens EJ, Kromhout D (1996). White blood cell count and the risk of coronary heart disease and all-cause mortality in elderly men. Arterioscler Thromb Vasc Biol.

[16] Ruggiero C, Metter EJ, Cherubini A, Maggio M, Sen R, Najjar SS, Windham GB, Ble A, Senin U, Ferrucci L (2007). White blood cell count and mortality in the Baltimore Longitudinal Study of Aging. J Am Coll Cardiol.

[17] Nilsson G, Hedberg P, Ohrvik J (2014). White blood cell count in elderly is clinically useful in predicting long-term survival. J Aging Res.

[18] Brown DW, Ford ES, Giles WH, Croft JB, Balluz LS, Mokdad AH (2004). Associations between white blood cell count and risk for cerebrovascular disease mortality: NHANES II Mortality Study, 1976-1992. Ann Epidemiol.

[19] Rosti G, Kopf B, Cariello A, Monti M, Dazzi C, Papiani G, Giovanis P, De Giorgi U, Marangolo M (2003). Prevention and therapy of neutropenia in elderly patients. Crit Rev Oncol Hematol.

[20] Repetto L, Biganzoli L, Koehne CH, Luebbe AS, Soubeyran P, Tjan-Heijnen VC, Aapro MS (2003). EORTC Cancer in the Elderly Task Force guidelines for the use of colony-stimulating factors in elderly patients with cancer. Eur J Cancer.

[21] (1997). https://wwwwhitehousegov/omb/fedreg_1997standards.

[22] Charlson ME, Pompei P, Ales KL, MacKenzie CR (1987). A new method of classifying prognostic comorbidity in longitudinal studies: development and validation. J Chronic Dis.

[23] Deyo RA, Cherkin DC, Ciol MA (1992). Adapting a clinical comorbidity index for use with ICD-9-CM administrative databases. J Clin Epidemiol.

[24] Huntley AL, Johnson R, Purdy S, Valderas JM, Salisbury C (2012). Measures of multimorbidity and morbidity burden for use in primary care and community settings: a systematic review and guide. Ann Fam Med.

[25] WHO (2011). Haemoglobin concentrations for the diagnosis of anaemia and assessment of severity. Vitamin and Mineral Nutrition Information System Geneva, World Health Organization, 2011 (WHO/NMH/NHD/MNM/111).

[26] Reagan JL, Castillo JJ (2012). Why is my patient neutropenic?. Hematol Oncol Clin North Am.

[27] Hsieh MM, Everhart JE, Byrd-Holt DD, Tisdale JF, Rodgers GP (2007). Prevalence of neutropenia in the U.S. population: age, sex, smoking status, and ethnic differences. Ann Intern Med.

[28] Reiner AP, Lettre G, Nalls MA, Ganesh SK, Mathias R, Austin MA, Dean E, Arepalli S, Britton A, Chen Z, Couper D, Curb JD, Eaton CB, Fornage M, Grant SF, Harris TB (2011). Genome-wide association study of white blood cell count in 16,388 African Americans: the continental origins and genetic epidemiology network (COGENT). PLoS Genet.

[29] Hershman D, Weinberg M, Rosner Z, Alexis K, Tiersten A, Grann VR, Troxel A, Neugut AI (2003). Ethnic neutropenia and treatment delay in African American women undergoing chemotherapy for early-stage breast cancer. J Natl Cancer Inst.

[30] Smith K, Wray L, Klein-Cabral M, Schuchter L, Fox K, Glick J, DeMichele A (2005). Ethnic disparities in adjuvant chemotherapy for breast cancer are not caused by excess toxicity in black patients. Clin Breast Cancer.

[31] Endres HG, Wedding U, Pittrow D, Thiem U, Trampisch HJ, Diehm C (2009). Prevalence of anemia in elderly patients in primary care: impact on 5-year mortality risk and differences between men and women. Curr Med Res Opin.

[32] Msaouel P, Lam AP, Gundabolu K, Chrysofakis G, Yu Y, Mantzaris I, Friedman E, Verma A (2014). Abnormal platelet count is an independent predictor of mortality in the elderly and is influenced by ethnicity. Haematologica.

